# Preclinical patient‐derived modeling of castration‐resistant prostate cancer facilitates individualized assessment of homologous recombination repair deficient disease

**DOI:** 10.1002/1878-0261.13382

**Published:** 2023-03-16

**Authors:** Mohamed E. Elsesy, Su Jung Oh‐Hohenhorst, Christoph Oing, Alicia Eckhardt, Susanne Burdak‐Rothkamm, Malik Alawi, Christian Müller, Ulrich Schüller, Tobias Maurer, Gunhild von Amsberg, Cordula Petersen, Kai Rothkamm, Wael Y. Mansour

**Affiliations:** ^1^ Department of Radiotherapy and Radiooncology University Medical Center Hamburg‐Eppendorf Germany; ^2^ Department of Tumor Biology, National Cancer Institute Cairo University Giza Egypt; ^3^ Martini‐Klinik Prostate Cancer Center University Medical Center Hamburg‐Eppendorf Germany; ^4^ Centre de Recherche du Centre Hospitalier de l'Université de Montréal (CRCHUM) QC Canada; ^5^ Mildred Scheel Cancer Career Center HaTriCS4 University Medical Center Hamburg‐Eppendorf Germany; ^6^ Department of Pediatric Hematology and Oncology University Medical Center Hamburg‐Eppendorf Germany; ^7^ Research Institute Children's Cancer Center Hamburg Germany; ^8^ Department of Molecular & Clinical Cancer Medicine University of Liverpool UK; ^9^ Bioinformatics Core University Medical Center Hamburg‐Eppendorf Germany; ^10^ Institute of Neuropathology University Medical Center Hamburg‐Eppendorf Germany; ^11^ Department of Urology University Medical Center Hamburg‐Eppendorf Germany; ^12^ Department of Oncology University Cancer Center Hamburg Eppendorf, University Medical Center Hamburg‐Eppendorf Germany

**Keywords:** castration‐resistant prostate cancer, *ex vivo* tumor cultures, homologous recombination, PARP inhibition, patient‐derived organoids

## Abstract

The use of mutation analysis of homologous recombination repair (HRR) genes to estimate PARP‐inhibition response may miss a larger proportion of responding patients. Here, we provide preclinical models for castration‐resistant prostate cancer (CRPC) that can be used to functionally predict HRR defects. *In vitro*, CRPC LNCaP sublines revealed an HRR defect and enhanced sensitivity to olaparib and cisplatin due to impaired RAD51 expression and recruitment. *Ex vivo*‐induced castration‐resistant tumor slice cultures or tumor slice cultures derived directly from CRPC patients showed increased olaparib‐ or cisplatin‐associated enhancement of residual radiation‐induced γH2AX/53BP1 foci. We established patient‐derived tumor organoids (PDOs) from CRPC patients. These PDOs are morphologically similar to their primary tumors and genetically clustered with prostate cancer but not with normal prostate or other tumor entities. Using these PDOs, we functionally confirmed the enhanced sensitivity of CRPC patients to olaparib and cisplatin. Moreover, olaparib but not cisplatin significantly decreased the migration rate in CRPC cells. Collectively, we present robust patient‐derived preclinical models for CRPC that recapitulate the features of their primary tumors and enable individualized drug screening, allowing translation of treatment sensitivities into tailored clinical therapy recommendations.

## Graphical abstract

Here, we demonstrated an enhanced sensitivity of acquired CRPC LNCaP sublines to olaparib or cisplatin due to lower RAD51 expression/loading. Olaparib or cisplatin increased the number of residual yH2AX/53BP1 foci in irradiated *ex vivo* slice cultures from CRPC patients. We further confirmed the HRR‐defect and enhanced olaparib or cisplatin sensitivity in CRPC patient‐derived tumor organoids (PDOs).

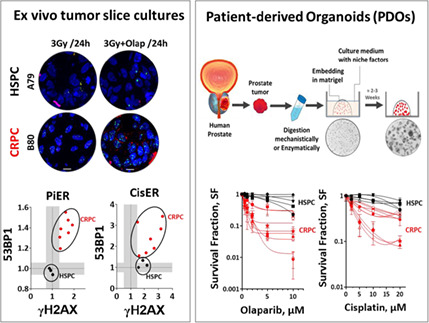

**Abbreviations**
Abi: abiraterone
ADT: androgen deprivation therapy
ARN: apalutamide
Bic: bicalutamide
CFA: colony formation assay
CisER: cisplatin‐induced enhancement ratio
COSMIC: catalog of somatic mutations in cancer
CR: castration‐resistant
CRPC: castration‐resistant prostate cancer
CSS: charcoal‐stripped serum
DAPI: 4′‐6‐diamidino‐2‐phenylindole
DMEM: Dulbecco's modified Eagle's medium
DSB: double strand break
DT: doubling time
EdU: 5‐ethynyl‐20‐deoxyuridine
ENZA: enzalutamide
H&E: hematoxylin and eosin
HP: hormone‐proficient
HRR: homologous recombination repair
mCRPC: metastatic castration‐resistant prostate cancer
mHSPC: metastatic hormone‐sensitive prostate cancer
MSI: microsatellite instability
MTT: 3‐(4, 5‐dimethylthiazolyl‐2)‐2, 5‐diphenyltetrazolium bromide
PARPi: PARP inhibitors
PCa: prostate cancer
PDO: patient‐derived tumor organoids
PiER: PARPi‐induced enhancement ratio
RNA‐SEQ: RNA‐sequencing
SBS: single base substitutions
SF: surviving fraction
WGS: whole genome sequencing

## 1 Introduction

Prostate cancer (PCa) is the most frequent malignant tumor in males; approximately one in six men will be diagnosed within their lifetime [1]. PCa is clinically variable, with often indolent and low‐risk disease that will not pose a health threat over one's lifetime, but also aggressive phenotypes with rapid disease progression and treatment resistance. In the metastatic stage of disease, patients have no option for cure despite significant progress with new therapeutic treatment strategies. Androgen deprivation therapy (ADT) induces tumor regressions in the vast majority of patients with metastatic hormone‐sensitive prostate cancer (mHSPC) and treatment intensification with docetaxel and/or androgen receptor (AR) pathway targeting agents has improved overall survival in randomized phase 3 clinical trials [2]. However, even at this early stage, the heterogeneity of the disease becomes obvious, with a nearly doubling of life expectancy in patients with metachronous development of metastases compared with patients with primary metastatic disease [3]. After systemic treatment initiation for mHSPC, virtually all patients progress to castration‐resistant prostate cancer (CRPC) as a result of selection and/or acquired resistance [4]. CRPC carries a worse prognosis, with estimated median survival times of 16–18 months from the onset of CRPC progression [5, 6, 7]. Although additional treatments exist for such patients, including docetaxel, enzalutamide, abiraterone, radium‐223, sipuleucel‐T and cabazitaxel, these treatment successes are not long‐lasting with only a modest overall survival benefit [8]. Comprehensive genomic characterization of CRPC identified frequent mutations in DNA repair genes, specifically those involved in homologous recombination repair (HRR) [9, 10], with a frequency reaching in some studies to approximately 40% among patients with metastatic CRPC (mCRPC) [11]. Consequently, the paradigm of PARP inhibitors (PARPi)‐mediated synthetic lethality or any other chemotherapy that targets HRR‐deficient tumors expands the management options for mCRPC. However, not all HRR defects respond equally to PARP inhibition. This led, for example, to a restricted approval of olaparib in Europe to patients with BRCA1/2 alterations, since the pivotal PROFOUND trial revealed the highest PARP inhibitor efficacy for patients carrying these respective mutations. In contrast, for alterations in other HRR genes, e.g. ATM, the effects were less convincing [12, 13]. The story gets even more complicated when comparing two recent phase 3 trials on the efficacy of a combination of abiraterone/prednisone and olaparib (PROpel) or niraparib (MAGNITUDE). Whereas in the PROpel trial, presumably due to a BRCAness effect of abiraterone, a prolonged progression‐free survival was also observed for the combination treatment in HRR wild‐type patients, the MAGNITUDE trial did not show such an advantage [14, 15]. This illustrates that we clearly do not yet comprehensively understand the molecular pathological processes of HRR in PCa. Furthermore, to date, HRR defects are basically detected by large‐scale sequencing analysis, a costly process. Despite these comprehensive analyses, information on the actual effect of these alterations on HRR function in the tumor cell is limited, which highlights the need for valid assays to functionally detect HRR defects.

Preclinical and translational research into novel synthetic lethality concepts is, however, hampered by the lack of appropriate preclinical models for such disease. Various preclinical models have been introduced to advance CRPC research. Most studies relied on using immortalized cell lines grown in two‐dimensional (2D) cultures or xenografts of such cell lines in immunocompromised animals. While these PCa cell lines are readily available and simple to use, only a limited number of cell lines are available and they are far from being authentic exemplars of CRPC due to their prolonged time in culture. In addition, the available cell lines fail to capture the various aspects of heterogeneity of PCa. Further, commonly used *in vitro* CRPC models, such as DU145 and PC3 cells, neither reflect the diversity of this disease, nor do they accurately predict patient sensitivity to treatment [16, 17]. Although several genetically engineered mouse models exist [18], they fail to generally model clinical CRPC, as castration‐resistant tumors in mice do not depend upon AR signaling mechanisms [19]. Therefore, three‐dimensional (3D) culture models of PCa are currently gaining increasing attention as preclinical models that better mimic the *in vivo* tumor biology and microenvironment. *Ex vivo* culturing of freshly collected tumor slices as well as patient‐derived organoids (PDOs) are considered promising 3D models. We and others have shown previously that tissue slice cultures show comparability with the original tumor, preserving the tumor morphology and its microenvironment [20, 21, 22, 23]. However, although the *ex vivo* assay allows a variety of functional analyses and biological readouts such as DSB repair and apoptosis, these do not include robust analysis of survival rates, one of the most important endpoints of drug sensitivity analysis.

Patient‐derived tumor organoids are 3D tissue cultures that promise to enable the validation of preclinical drug testing in precision medicine and co‐clinical trials by modeling tumors for predicting therapeutic responses with more reliable efficacy. Although there has also been a significant improvement in the generation of PDOs from PCa patients, their long‐term propagation in culture has remained challenging. To the best of our knowledge, there are only a few studies reporting successful establishment of PDOs from PCa specimens [24, 25, 26, 27, 28, 29], with a success rate of < 20%. Furthermore, the capacity for long‐term maintenance of these PDOs is variable and limited [30]. In the current study, we provide different preclinical models derived from CRPC *in vivo* and the rationale for using these models to recapitulate and predict the response of individual CRPCs to PARP inhibitors, that is, olaparib and cisplatin.

## 2 Materials and methods

### 2.1 Cell culture, drugs and X‐irradiation

LNCaP prostate cancer cells (ATCC, Manassas, VA, USA) were grown in Dulbecco's modified Eagle's medium (DMEM, Gibco,  Paisley, UK) supplemented with 10% fetal calf serum (FCS), 100 U·mL^−1^ penicillin and 100 mg·mL^−1^ streptomycin at 37 °C with 10% CO_2_. Novel antiandrogen‐resistant sublines LNCaP‐ARN509 and LNCaP‐abi were generated by long‐term treatment with apalutamide (ARN‐509) (up to 40 μM) and abiraterone acetate (up to 10 μM), respectively, until acquiring androgen‐independent growth feature [31]. C4‐2B‐Enza cells (kindly provided by Prof. C. P. Evans, UC Davis School of Medicine, Sacramento, CA, USA) were maintained in medium containing 20 μM enzalutamide. Abiraterone acetate, apalutamide and enzalutamide were kindly provided by Janssen Cilag GmbH, Neuss, Germany. LNCaP‐abl cells (a gift from Prof. Z. Culig, Medical University Innsbruck, Austria) were grown in DMEM supplemented with 10% Charcoal Stripped FBS (Sigma‐Aldrich, Taufkirchen, Germany) [32]. All cell lines tested negative for mycoplasma contamination. Irradiation was performed as previously described (200 kV, 15 mA, additional 0.5‐mm Cu filter at a dose rate of 0.8 Gy·min^−1^) [15].

### 2.2 Cell lines authentication

All cell lines used in the current study have been authenticated before executing the experiments. Authentication of cell lines used in the current study was performed in our laboratory. The profiles for all cell lines have been compared and matched with the STR profile database.

### 2.3 Proliferation assay

Proliferation assay was performed as previously described [31]. Briefly, cells were cultured in triplicate in 6‐well plates before treatment. For LNCaP‐abl subline, cells were seeded in CS‐FCS‐supplemented medium for 18–24 h before changing to the FBS full medium for the treatment. To assess the effect of any treatment regimes, the cell number was determined with a Beckman Coulter cell counter (Life Science, Beckman Coulter cell counter, Krefeld, Germany) at 3‐, 6‐ and 10‐days post‐treatment. In all experiments, media with or without drugs were changed twice during the 10‐day treatment course.

### 2.4 3D colony formation assay

3D colony formation assay (CFA) for cell lines was performed by mixing cells in cold‐reduced growth factor basement membrane extract (RGF BME) type 2 (R&D Systems, Minneapolis, MN, USA) and platted at 2000 cells per dome onto 24‐well plates. For tumor organoids 3D CFA, tumor organoids were first harvested and sheared into single cells before being mixed in cold BME and plated at 4000 cells per dome. Upon completed gelation, different concentrations of olaparib or cisplatin as well as DMSO controls were added in triplicate in 500 μL of corresponding medium. After 3–4 weeks, colonies (tumoroids or 3D cell cultures) were stained with 0.5 mg·mL^−1^ 3‐(4,5‐dimethylthiazolyl‐2)‐2, 5‐diphenyltetrazolium bromide (MTT) for 1.5 h. For organoid colonies, medium was removed and BME domes were dissolved using Cultrex™ Organoid Harvesting Solution. MTT‐stained 3D cellular colonies and tumoroids were photographed using REBEL Microscopy (ECHO, San Diego, CA, USA) and analyzed using image‐j. Surviving fractions (SFs) were calculated by normalization to the plating efficiency of the untreated control. DMSO was used as a control at the same concentration.

### 2.5 Immunofluorescence

After treatment, cells cultured on coverslips were washed and fixed with 4% paraformaldehyde/PBS for 10 min. Fixed cells were permeabilized with 0.2% Triton X‐100/PBS on ice for 5 min and incubated for 1 h at room temperature with primary antibodies: mouse monoclonal anti‐phospho‐S139‐H2AX antibody (Millipore, Darmstadt, Geramay) at a dilution of 1 : 500 and rabbit polyclonal anti‐ 53BP1 antibody (Novus, Braunschweig, Germany) at a dilution of 1 : 500 or rabbit polyclonal anti‐RAD51 (Sigma Aldrich, Taufkirchen, Germany ) at a dilution of 1 : 500. After being washed three times with cold PBS, the cells were incubated for 1 h with secondary anti‐mouse Alexa Fluor 594 (Invitrogen) at a dilution of 1 : 500, and anti‐rabbit Alexa Fluor 488 (Invitrogen) at a dilution of 1 : 600. The nuclei were counterstained with 4′‐6‐diamidino‐2‐phenylindole (DAPI, 10 ng·mL^−1^). Slides were mounted in Vectashield mounting medium (Vector Laboratories, Newark, CA, USA). Immunofluorescence of cultured tumor tissue was performed as previously described [21]. Fluorescence microscopy was performed using the Zeiss AxioObserver.Z1 microscope (objectives: ×20, resolution 0.44 μm; Plan Apo 63/1.4 Oil DICII, resolution 0.24 μm; and filters: Zeiss 43, Zeiss 38, Zeiss 49, Göttingen, Germany). Z‐stacks of semi‐confocal images were obtained using the zeiss apotome, zeiss axiocam mrm and zeiss axiovision software. For DSB analysis, fields of view were taken per time point or treatment with a minimum of 100 cells (cell lines) or 50 cells (tumor tissue). All staining was performed in duplicate. DSBs were analyzed using image‐j and DAPI‐based image masks and normalized to single nucleus values [21].

### 2.6 Western blot

Whole cell lysates were subjected to western blot as previously described [33, 34]. RAD51 immunoblot analysis was performed with the rabbit anti‐RAD51 (Merck, Darmstadt, Germany, Cat#PC130). Beta‐actin was immunoblotted by mouse anti‐beta‐actin (Sigma Aldrich, Taufkirchen, Germany, Cat#A1978) and used as a loading control. Goat‐anti‐mouse IgG‐Alexa Fluor 594 (Molecular Probes, Sigma Aldrich, Taufkirchen, Germany, Cat#A11005) and goat‐anti‐rabbit IgG‐AlexaFluor 488 (Molecular Probes, Cat#A11008) secondary antibodies were used. Membranes were developed and analyzed using LiCor Biosciences (Lincoln, NE, USA) at room temperature.

### 2.7 Patient sample collection

Fresh PCa tissue was obtained from patients with high‐risk PCa according to D'Amico risk stratification, who underwent radical prostatectomy at Martini‐Klinik, Prostate Cancer Center Hamburg, Germany between 2019 and 2022. Immediately after resection, one to two punch biopsies were taken by the surgeon in palpable tumor areas. The biopsies were collected in culture media and immediately taken to the laboratory. Pseudonymized biopsies were processed within 30 min after resection. The laboratory received a final pathology report containing the Gleason score, PSA status and age of each pseudonymized patient for clinical analysis. The project was approved by the local ethics committee [Ethik‐Kommission der Ärztekammer Hamburg] with the project number PV7007. The study methodologies conformed to the standards set by the Declaration of Helsinki. All experiments were undertaken with the understanding and written consent of each subject.

### 2.8 Tissue slice cultures

*Ex vivo* tissue slice cultures were prepared as previously described [20]. Briefly, 300‐μm slices were cut using the MacIllvine tissue chopper and placed on Millicell® cell culture inserts (0.4 μm, 30 mm diameter, Merck), which were inserted in 6‐well dishes containing 1 mL DMEM supplemented with 10% FCS and incubated at 37 °C. Prior to *ex vivo* treatment, the tissues slices were incubated for 1 day for recovery and re‐oxygenation. To monitor proliferation, un‐irradiated slice cultures were incubated with 5‐ethynyl‐20‐deoxyuridine (EdU, 1 : 1000; Click‐iT Assay Kit, Invitrogen) overnight for 16 h. All slices were additionally treated with pimonidazole (200 μM, Hypoxyprobe, Burlington, MA, USA) 2 h before fixation to monitor tissue hypoxia.

### 2.9 Histology and imaging

PCa tumor tissues and PDOs were prepared as previously described [21]. Briefly, either tissues or tumoroids were fixed using 4% PFA (Merck) followed by washing in 25% sucrose twice each for 1 h. The samples were then frozen in TissueTek® (Sakura Finetek, Alphen aan den Rijin, Netherlands) and stored at − 80 °C. Using the Cryo Star NX70 Microtome (Thermo Scientific) sectioning was performed to prepare cryoslices (5 μm). Histological analysis was performed by standard hematoxylin and eosin (H&E) staining and percentage of cancer cells and Gleason score were determined by an experienced PCa pathologist. Immunohistochemistry was performed using antibodies against AMACR (Thermo Scientific, Regensburg, Germany, PA5‐82739, 1 : 250), and Ki67 (Abcam, Cambridge, UK, ab15580, 1 : 250). Images were acquired using ZEISS Axio Scan.Z1 Slide Scanner and photos were then processed using netScope® Viewer.

### 2.10 Prostate tumor tissue processing and organoid establishment

Organoid establishment and culture were adapted from Drost et al. [25] and Gao et al. [30]. Briefly, prostate tumor specimens from patients who underwent radical prostatectomy were received in adDMEM F12+++ [advanced DMEM F12 (Thermo Scientific) supplemented with 10 mm HEPES (Thermo Scientific), GlutaMAX (Thermo Scientific), and penicillin/streptomycin (Thermo Scientific)]. Tumor tissue samples were first washed three times with PBS and then placed in 3.5‐cm culture dish where they underwent mechanical dissection into small pieces, which were then placed in 5 mg·mL^−1^ Collagenase type II (Sigma‐Aldrich) in adDMEM +++ with 10 μM ROCK inhibitor and incubated in a 37 °C shaker for 30–90 min for digestion. After tissue digestion, the suspension was passed through a 50‐μm cell strainer (Sysmex) before being washed with adDMEM F12 +++ and finally suspended in cold BME. Drops of BME cell suspension each 40 μL were allowed to solidify for 30 min at 37 °C onto a pre‐warmed 24‐well culture plate. After stabilization of Matrigel‐containing cells, 500 μL of complete organoid medium (Table S1) was added. Fresh medium was replenished every 3–4 days during organoid growth. Organoids were passaged every 4–6 weeks. During passaging, the organoid droplets were mechanically sheared through P1000 pipet tip and incubated with TrypLE Express containing 10 μM ROCK inhibitor for a maximum of 5 min at 37 °C. The resulting cell clusters and single cells were washed and re‐plated following the protocol described above.

### 2.11 *Ex vivo* induction of castration‐resistant status

To induce castration‐resistant phenotype *ex‐vivo*, PCa tissues derived from naïve PCa patients were cultured in hormone‐depleted condition (DMEM supplemented with CS‐FBS containing 10 μm abiraterone). Each sample was cultured for up to 6 weeks in either hormone‐proficient or ‐deficient conditions and the culture medium was refreshed every 3 days. The proliferative marker Ki67 was used to prove the attained castration‐resistant phenotype through proliferative activity. The tumor tissue cultured in hormone‐deficient condition that still showed Ki67 proliferative index similar to its counterpart slice cultured in hormone proficient medium, was considered a castration‐resistant sample.

### 2.12 Migration assay

Chemotaxis assay was performed in 24‐well Transwell plate using 8‐μm pore‐size (Corning^®^ BioCoat^®^, 354578). Either LNCaP or LNCaP‐ARN509 cells were harvested and re‐suspended in FBS‐free DMEM medium at concentrations of 2 × 10^5^ cells in 0.2 mL, and then seeded into the upper chamber of a 24‐well plate. The lower chambers were filled with 0.7 mL DMEM containing 10% FBS to act as an attractant. Cells were incubated for 36 h. At the end of the experiment, cells that migrated into the reverse side of the Transwell membrane were fixed with 70% ethanol, stained with 0.2% crystal violet, and then photographed using REBEL Microscopy and analyzed using image‐j.

### 2.13 DNA methylation profiling

Total DNA was isolated from PCa cell lines, tissues or organoid cultures using Qiagen Kit (Qiagen, Hilden, Germany). The Illumina HumanMethylation450 BeadChip (450 K) arrays were used to analyze genome‐wide DNA methylation patterns of tissues or organoids. Only sites covered by at least three reads were considered for analysis. For each sample, the percentage of methylation per site (beta value) was computed. Average hierarchical clustering of samples was performed by ‘1‐Pearson's correlation coefficient’ as distance measured on the *n* = 10 000 CpG sites showing the highest standard deviation across the cohort. Several samples of the following datasets were included as a reference set: TCGA‐BRCA, TCGA‐Lung, TCGA‐GBM, TCGA‐PRAD, GSE112047, GSE38240, GSE83842 and glioblastoma samples (tissue and cell lines) and lung carcinoma samples were from UKE.

### 2.14 Whole genome sequencing (WGS)

WGS was performed by Novogene (Sacramento, CA, USA). WGS data analysis was performed by the Bioinformatics core facility at University Medical Center Hamburg‐Eppendorf (UKE), Hamburg, Germany. Reads were aligned to the human genome assembly GRCh38 using bwa mem [35] and structural and short somatic mutations were labeled using Manta [36] and Strelka2 [37], respectively. Variants with a depth below 60 or presence in the Genome aggregation database (https://gnomad.broadinstitute.org/) were removed. Single base substitutions (SBS) – as defined by the Catalog of Somatic Mutations in Cancer (COSMIC) – were identified using sigProfiler [38] and putative microsatellite instability (MSI) was determined using MANTIS [39]. The method HRDetect described by Davies et al. [40] for the identification of homologous recombination deficiency was applied using the R package signature.tools.lib [41].

### 2.15 RNA‐sequencing (RNA‐SEQ)

Total RNA was extracted from PCa cells using RNeasy Mini Kit (Qiagen, Hilden, Germany). RNA were then sent to Novogene for RNA‐SEQ libraries preparation and sequencing. RNA‐SEQ data analysis was performed by Novogene. Briefly, reads were aligned to the human reference genome GRCh37 using STAR [42] and differential expression analysis between LNCaP cells and their castration‐resistant sublines was performed using r package edger [43]. Genes with a false discovery rate < 0.005 and an absolute log2‐fold‐change > 1 were considered significant. Enrichment analysis of Gene ontology terms and pathway categories was carried out using r package clusterprofiler [44].

### 2.16 Graphs and statistics

Statistical analyses, data fitting and graphics were performed with the graphpad prism 9.0 program (GraphPad Software, Boston, MA, USA). The IDAT files of the samples were loaded, filtered and normalized with the package limma (version 3.40.0) in r (version 3.6.0). By using multiple datasets containing different numbers of CpG sites, our samples were reduced to 450 k sites. In addition, a correction was made for possible batch effects related to chip size using the limma package.

## 3 Results

### 3.1 Castration‐resistant cells are more radiosensitive than hormone‐sensitive cells due to impaired DSB repair

**Fig. 1:**
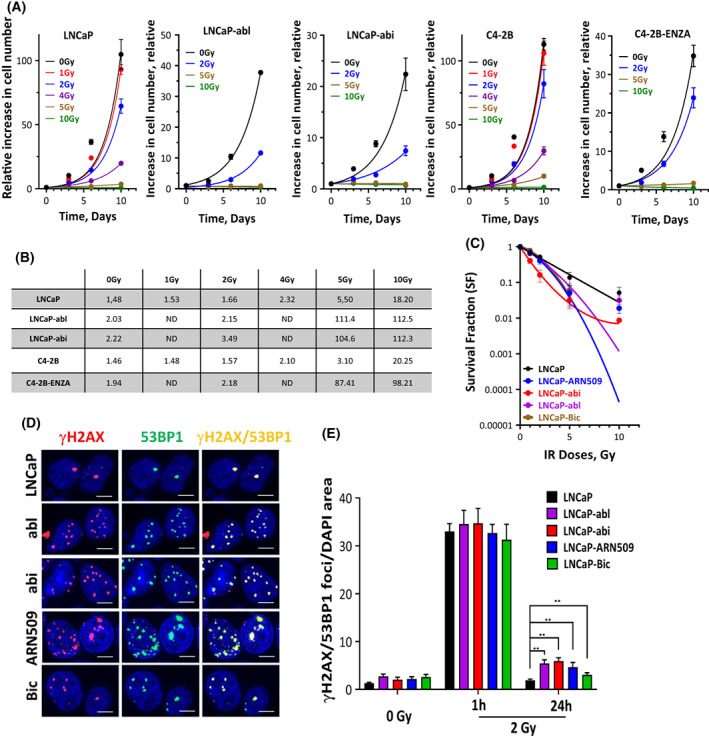
Castration‐resistant cells are more radiosensitive than the parental hormone‐sensitive cells. (A) Cell number was determined in LNCaP, LNCaP‐abl, LNCaP‐abi, C4‐2B and C4‐2B‐ENZA cells on days 0, 3, 6 and 10 post‐irradiation with the indicated doses. (B) Cell doubling time in days was calculated for each treatment by fitting exponential growth curves using graphpad prism 9. Shown are means ± SEM of three independent experiments. (C) Radiosensitivity of the indicated cells was analyzed by colony formation assay to calculate survival fractions after different irradiation doses. Shown are means ± SEM of four independent experiments. (D) Representative micrographs of γH2AX/53BP1 foci (Scale bar: 100 μm) in the indicated cells 24 h after irradiation with 2 Gy and (E) quantifications of γH2AX/53BP1 foci in the indicated cells before and 1 and 24 h after irradiation with 2 Gy. Shown are means ± SEM of three independent experiments. Significance was calculated using the Mann–Whitney *U*‐test: **P < 0.01 vs. control.

Previously, we reported that DSB repair in CRPC cells is less efficient than in hormone‐sensitive cells [31]. Since DSB repair capacity is a determinant factor for cellular radiosensitivity [45], we sought to analyze radiosensitivity in CRPC cells. To that end, LNCaP cells and their castration‐resistant sublines (LNCaP‐abl, LNCaP‐abi, C4‐2B, and C4‐2B‐ENZA) were treated with different IR doses and the effect on cell growth was monitored by cell counting at 3‐, 6‐ and 10‐days post‐irradiation. A remarkable irradiation‐related decrease in cell growth was observed in the resistant clones compared with the parental cells (Fig. 1A). Consistently, IR resulted in a significant increase in doubling times (DT) in resistant clones compared with their parental cell lines (Fig. 1B), indicating an enhanced radiosensitivity in CRPC sublines. In keeping with this idea, using Matrigel‐based 3D‐culturing, we identified a significant radiosensitivity in all CRPC clones compared with their sensitive parental cells using colony forming assay (Fig. 1C). To analyze DSB repair efficiency, androgen‐sensitive LNCaP and castration‐resistant sublines were exposed to IR with a dose of 2 Gy, and γH2AX and 53BP1 foci were quantified after 1 and 24 h post‐irradiation (Fig. 1D). Although we observed no difference in the number of γH2AX/53BP1 between sensitive and resistant cells at the 1‐h time point post‐2 Gy, the exposure to 2 Gy significantly increased the number of residual γH2AX/53BP1 foci (threefold) at 24 h in all resistant sublines compared with sensitive LNCaP cells, pointing at impaired double‐strand break repair capacity (Fig. 1E).

### 3.2 Impaired HR in castration‐resistant cells due to lower RAD51 expression and loading

**Fig. 2:**
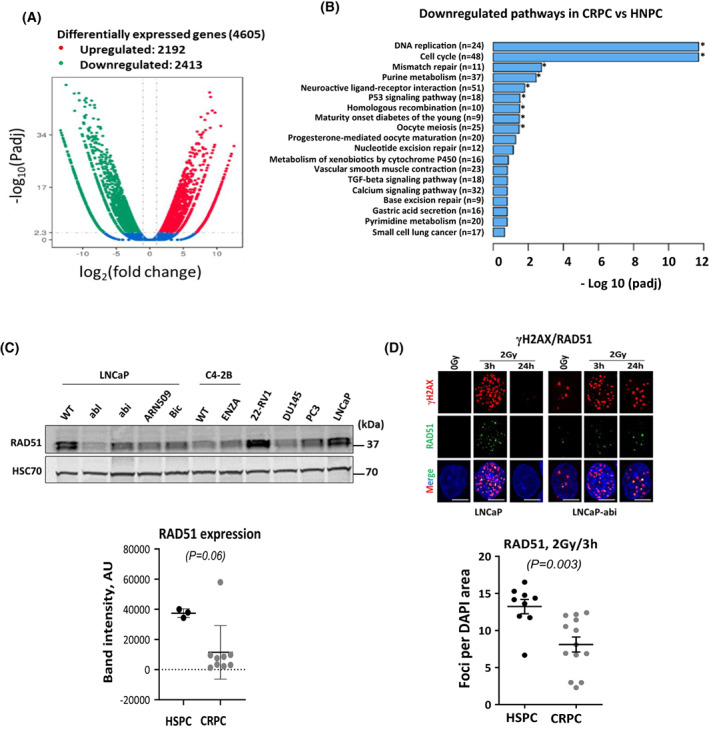
Castration‐resistant cells show a HRR deficiency due to RAD51 downregulation. (A) Volcano plot showing differentially expressed genes in polled castration‐resistant sublines (*n* = 4) vs. hormone‐sensitive LNCaP cells (*n* = 2), measured by RNA‐SEQ. (B) Gene ontology (GO) enrichment analysis of the significantly downregulated pathways [−log10 (adjusted *P* value)] in castration‐resistant cells. Significance: *P < 0.05. (C) Upper panel: Western blotting showing the expression of RAD51 protein in LNCaP cells (WT), castration‐induced LNCaP sublines (abl, abi, ARN509, Bic), *in vivo* induced castration‐resistant C4‐2B cells and their enzalutamide‐resistant subline (Enza), as well as 3 castration‐resistant cell lines (22‐RV1, DU145, and PC3). HSC70 was used as a loading control. Lower panel: band intensities were calculated from three independent blots. (D) Upper panel: Representative immunofluorescence images of γH2AX and RAD51 foci (Scale bar: 100 μm) detected after 3 and 24 h post‐irradiation with 2 Gy. DAPI counterstain was used to visualize nuclei. Lower panel: Quantification of RAD51 foci numbers induced at 3 h post irradiation with 2 Gy. Shown are means ± SEM of three independent experiments. Significance was calculated using the Mann–Whitney *U*‐test vs control.

To unveil the mechanism underlying the impaired DSB repair in castration‐resistant cells, we compared the transcription profile of LNCaP cells and their castration‐resistant sublines using RNA‐SEQ in biological duplicates. We then pooled all resistant clones and compared the commonly expressed genes with those in their parental hormone‐sensitive LNCaP cells. More than 4500 genes were found to be significantly differentially expressed in the resistant clones, 2413 genes of which were downregulated in pooled resistant clones (Fig. 2A). Interestingly, gene ontology analysis (Fig. 2B) revealed that the most differentially repressed molecular pathways were DNA damage‐response pathways including DNA replication, cell cycle and HRR. Among the HR repressed genes, RAD51 was significantly downregulated in resistant sublines (https://www.ebi.ac.uk/ena/browser/view/PRJEB55017). Given that the level of RNA is not necessarily always correlated with protein levels, we analyzed RAD51 protein levels in LNCaP cells and their castration‐resistant sublines as well as in 22‐RV1, DU145 and PC3 cell lines, which have been established from xenografts or metastatic lesions of patients with CRPC. Except for the 22‐RV1 cells, twofold lower RAD51 protein levels were detected in resistant LNCaP sublines as well as in other castration‐resistant cells than in hormone‐sensitive LNCaP cells (Fig. 2C), indicating impaired HR in castration‐resistant cells. To recapitulate this, LNCaP cells and their resistant sublines were irradiated with 2 Gy and RAD51 colocalized with γH2AX foci were monitored at 3 and 24 h post‐irradiation (Fig. 2D, upper panel). As illustrated in the lower panel of Fig. 2D, the number of RAD51/γH2AX foci was significantly lower (twofold) in resistant clones than in sensitive LNCaP cells (*P* = 0.003). Together, these data indicate that HRR may be impaired during transition to a castration‐resistant phenotype.

### 3.3 Castration‐resistant cells are more sensitive to olaparib or cisplatin

**Fig. 3:**
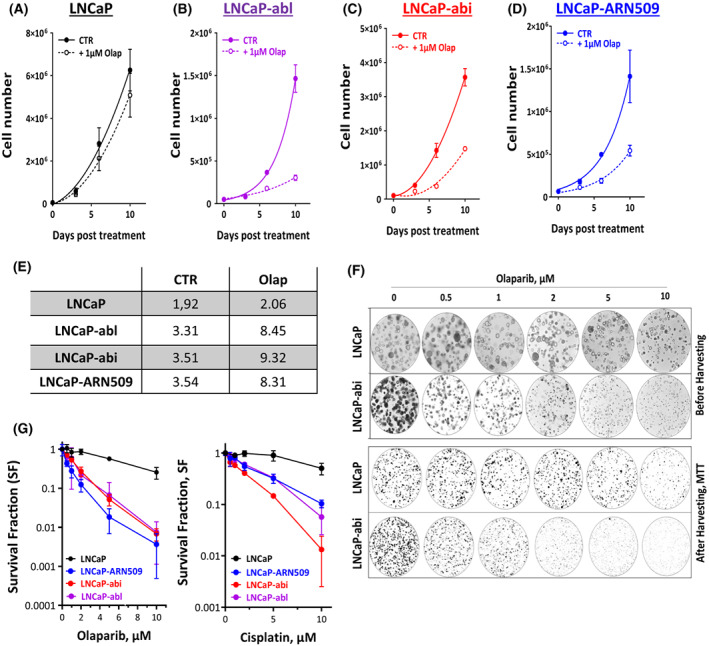
Olaparib is more toxic for castration‐resistant cells than for the parental hormone‐sensitive cells. Cell number was determined in LNCaP (A), LNCaP‐abl (B), LNCaP‐abi (C) and LNCaP‐ARN509 (D) cells on days 0, 3, 6 and 10 post treatment with 1 μM of the PARP inhibitor olaparib. (E) Cell doubling time in days was calculated for each treatment by fitting exponential growth curves using graphpad prism 9. Shown are means ± SEM of four independent experiments. (F) Representative images of 3D‐cultures of the LNCaP and LNCaP‐abi cells treated with the indicated concentrations of olaparib before (upper panel) and after (lower panel) harvesting and staining with MTT. (G) Survival fractions measured by colony forming assay after treating the indicated cells with different concentrations of olaparib (left panel) or cisplatin (right panel). Shown are means ± SEM of three independent experiments.

A direct association between the response to PARPi or cisplatin and non‐functional HRR pathway was reported [19, 32]. We therefore investigated the sensitivity of castration‐resistant cells to PARP inhibition with olaparib. To this end, the effect of 1 μM olaparib on the proliferation of castration‐resistant cells was measured. Although olaparib did not reduce the cell growth in the hormone‐sensitive LNCaP cells (Fig. 3A), it significantly decreased the proliferation rate in LNCaP‐abl (Fig. 3B), LNCaP‐abi (Fig. 3C) and LNCaP‐ARN509 (Fig. 3D), as exemplified by an increase in DT (1.3‐, 2.6‐ and 2.3‐fold, respectively; Fig. 3E). To further verify this, LNCaP and resistant sublines were treated with different concentrations of olaparib (0, 0.5, 1, 2, 5 and 10 μM) and effects on survival were analyzed using 3D Matrigel CFA (Fig. 3F). To ensure better visualization and counting of living cells, MTT was used to stain living cells within 3D cultures pre‐harvesting. Again, a significantly enhanced sensitivity to olaparib was observed in castration‐resistant sublines compared with parental LNCaP cells (Fig. 3G). Similar results were obtained for cisplatin (Fig. 3G).

Since CRPC is a very heterogeneous disease, we sought to analyze the genome profile of the CR LNCaP sublines to elucidate whether they carry the alterations found in CRPC *in vivo*. WGS data reported no big structural differences in CRPC sublines compared with their hormone naïve parental cells (Fig. S1A). Mutational signatures analysis revealed that single base substitutions (SBS) – as defined by the Catalog of Somatic Mutations in Cancer (COSMIC) – detected in the CR samples included age‐related signatures SBS1 and SBS5 but also SBS44, which is associated with defective DNA mismatch repair (Fig. S1B). In fact, all CR samples had high MSI scores (LNCaP‐ARN509: 0.69, LNCaP‐abi: 0.72, LNCaP‐abl: 0.69, LNCaP‐bic: 0.73), indicating genomic instability in the derived CRPC sublines. No mutation signature for HRR defect was detected in CR cells, despite showing a functional HRR deficiency associated with lower RAD51 expression at the transcriptional level. Possibly, CRPC sublines did not have enough time during establishment of resistance phenotype to accumulate genetic aberrations to show the HRR defect signature.

### 3.4 *Ex vivo* induction of castration resistance as an approach to study the sensitivity of CRPC to olaparib or cisplatin

**Fig. 4:**
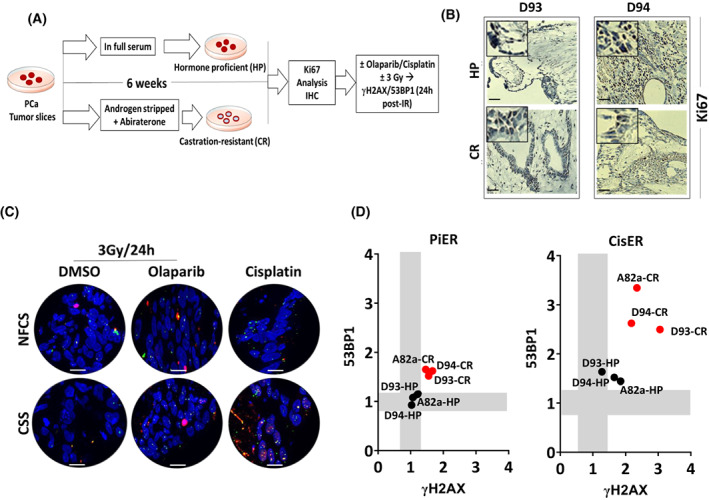
*Ex vivo* induction of castration resistance in prostate cancer increases cytotoxicity to olaparib and cisplatin via impairing double strand break repair efficiency. (A) Schematic representation of castration resistance induction in *ex vivo* cultures of prostate cancer (PCa) specimens which had shown no signs of castration resistance *in vivo*. Briefly, PCa slices were cultured for up to 6 weeks in either androgen‐stripped serum containing medium in the presence of abiraterone (10 μM) or in full serum containing medium. Ki67 expression was monitored by immunohistochemical staining. Only tumor slices showing a similar Ki67 index under castration resistance culturing conditions were included in the next experiments. (B) Representative images of Ki67 staining (Scale bar: 100 μm) in two tumor slices from two PCa patients (D93 and D94) cultured under hormone‐proficient (HP) or castration resistance‐inducing (CR) conditions. High magnification images (magnification, 80×) of ROI are shown. (C) Representative micrographs of γH2AX (red) and 53BP1 (green) foci 24 h after treatment with olaparib plus 3 Gy or cisplatin alone in PCa slices from patient #D93. Scale bar: 100 μm. (D) Plots showing the correlation between PARP inhibitor (PARPi) enhancement ratio (PiER, left panel) or cisplatin enhancement ratio (CisER, right panel) of residual γH2AX (*X*‐axis) and 53BP1 (*Y*‐axis) at 24 h post treatment; three independent experiments for each tumor slice. Black and red dots represent tumor slices cultured under hormone‐proficient and castration resistance conditions, respectively.

The above results may imply that HRR is compromised during the development of castration resistance. To further confirm this hypothesis, we used an approach to *ex vivo* inducement of a castration‐resistant phenotype in primary hormone naïve prostate tumor specimens (Fig. 4A). Briefly, tumor slices from three patients with hormone naïve prostate cancer were cultured *ex vivo* for up to 6 weeks in androgen‐depleted medium supplemented **with** charcoal‐stripped serum (CSS) in the presence of abiraterone to induce a CRPC state. Ki67 IHC staining was used to confirm the development of the androgen‐independency of the cultured primary tumor samples. Tumor slices showing no change in Ki67 index after culturing under castration‐resistant (CR) conditions were considered to be CRPC. As a control, tumor slices from the same three patients were cultured under hormone‐proficient (HP) conditions, i.e. in normal medium containing FCS plus DHT for the same period. We assessed this approach with 12 PCa samples from individual eight PCa patients, but only three samples from three patients showed no difference in the Ki67 index upon hormone‐depleted culturing conditions (Fig. 4B). Next, we compared the radiosensitization effect mediated by either olaparib or cisplatin in PCa tissue slices cultured under either HP or CR conditions. To that end, slice cultures from more than six tumor punch biopsies collected from 3 PCa patients were irradiated *ex vivo* with 3 Gy in the presence or absence of either olaparib or cisplatin. γH2AX and 53BP1 foci were then analyzed 1 and 24 h later (Fig. 4C). No change was observed in the number of foci at the 1‐h time point between the slices cultured under either condition. However, tumor slices cultured in CR conditions demonstrated an increased number of residual γH2AX and 53BP1 at 24 h post‐IR (Fig. S2A,B). Further, the radiosensitization enhancement ratio mediated by olaparib (PiER) or cisplatin (CisER) was evaluated using the mean standard error of all samples set as a threshold for each DSB marker. Data clearly showed that the PiER of tumor slices cultured under CR but not HP rose  above the threshold, with minor alterations between both markers (Fig. 4D). This confirms the findings in Fig. 3 that HRR is compromised in the induced CR models.

### 3.5 Olaparib increases the cytotoxicity of ionizing radiation in castration‐resistant but not hormone naïve prostate cancer tissues

**Fig. 5:**
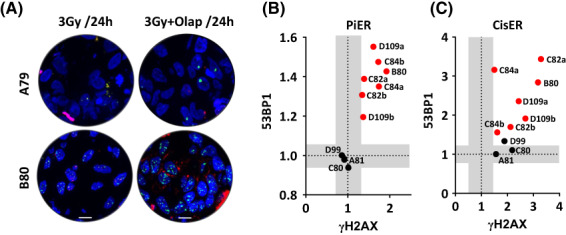
Olaparib or cisplatin increases cytotoxic effects of IR in castration‐resistant PCa. (A) Representative micrographs of γH2AX (red) and 53BP1 (green) foci 24 h after treatment with olaparib plus 3 Gy in tumor biopsies from a castration‐resistant PCa patient #B80. Scale bar: 100 μm. (B) PiER of residual γH2AX (*X*‐axis) and 53BP1 (*Y*‐axis) foci at 24 h post 3 Gy. (C) Cisplatin‐enhancement ratio (CisER) of residual γH2AX (*X*‐axis) and 53BP1 (*Y*‐axis) at 24 h. Shown are mean ± SEM of three independent experiments. Black and red dots represent data from hormone naïve and castration‐resistant PCa, respectively.

To further validate the above findings and the applicability of the presented preclinical CRPC models, we sought to recapitulate the data from Fig. 4, using tumor biopsies collected from CRPC patients. A total of 13 tumor biopsies from nine PCa patients (CRPC; *n* = 5 and HSPC; *n* = 4) were collected and irradiated *ex vivo* with 3 Gy after treatment with either olaparib or cisplatin. The impact of irradiation on the number of γH2AX/53BP1 foci was then analyzed 1 and 24 h later (Fig. 5A) and the PiER and CisER were assessed. Ultimately, 10 punch biopsies from seven patients fulfilled all the previously described requirements [21] and were therefore used for further analysis. Again, no difference was observed in the number of γH2AX/53BP1 foci at 1 h post‐3 Gy between CRPC and HSPC biopsies; however, tumor biopsies from CRPC patients showed a distinct increase at 24 h for both γH2AX and 53BP1 markers upon pretreatment with olaparib (Fig. S3A,B). Consistently, PiER of all biopsies from CRPC clearly increased above the threshold, again with minor alterations between both DSB markers (Fig. 5B). Similar results were obtained using cisplatin (Fig. S3A,B, Fig. 5C). Together these findings reveal the plausibility of the preclinical models of CRPC to detect DSB repair defect, sensitivity to cisplatin and radiosensitization effect of olaparib.

### 3.6 Establishment and characterization of patient‐derived organoids cultures from PCa patients

**Fig. 6:**
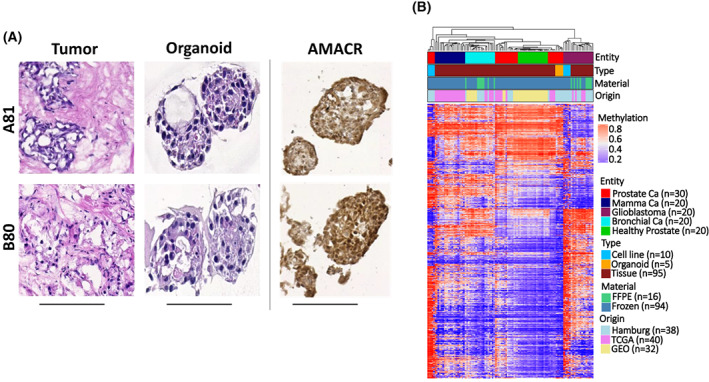
Establishment and characterization of patient‐derived organoid cultures from prostate cancer (PCa) patients. (A) Representative images of the established organoids and their corresponding primary tumors with H&E and immunohistochemical staining for AMACR. Scale bar: 50 μm. (B) Genome‐wide DNA methylation cluster analysis of the established organoids showing clustering with PCa but not with normal prostate or other tumor entities using a cohort of 110 samples.

Twelve long‐term PDO cultures from four naïve and four CRPC patients were established using a modified protocol from Gao et al. [30]. We obtained a success rate of more than 60%. All established PDOs were successfully expanded and maintained under the same culturing conditions for > 12 passages with no obvious morphological changes (data not shown) and were frozen down to create an organoid biobank. Clinical information and pathological parameters showed similarities between the established patient‐derived organoids and their donors (Table S2). The histological features of each of the established PDOs were of similar appearance to their matched primary tumors (Fig. 6A), with a strong AMCAR immunohistochemistry staining (Fig. 6A). We also evaluated CpG‐rich methylation in PDOs on a genome‐wide scale using Illumina HumanMethylation450 Bead Chip (450 K) arrays [46]. The PDOs models were found to cluster with PCa but not with normal prostate or other tumor entities based on DNA methylation profiling (Fig. 6B). Interestingly, the methylation profile of the PDOs did not cluster with that of the established cell lines, indicating that the PDOs more appropriately represent the patients' *in vivo* tumor. Altogether, this confirms that the established PDOs represent the PCa *in vivo* and can therefore serve as preclinical models for PCa research.

### 3.7 Olaparib and cisplatin are more toxic for organoids established from CRPC than hormone naïve patients

**Fig. 7:**
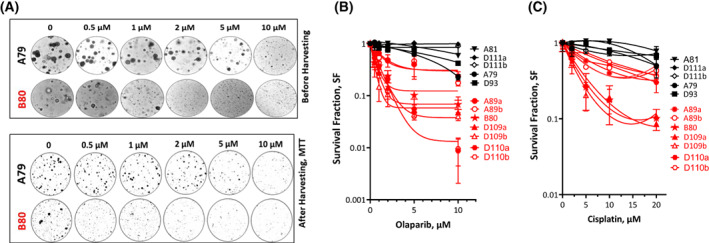
Olaparib is more toxic for castration‐resistant organoids than for the hormone‐sensitive PCa organoids. (A) Representative images of organoid cultures of a hormone naïve (A79) and castration‐resistant (B80) PCa organoid treated with the indicated concentrations of olaparib before (upper panel) and after (lower panel) harvesting and staining with MTT. (B,C) Survival fractions measured by colony forming assay after treatment of organoids established from hormone naïve (black) or castration‐resistant (red) PCa patients with different concentrations of olaparib (B) or cisplatin (C). Shown are means ± SEM of four independent experiments (except for hormone näive patients, where *n* = 3).

The advantage of using PDOs as preclinical models is that they not only allow DSB repair monitoring but also enable the analysis of the effect of olaparib or cisplatin on clonogenic survival by colony formation assay. Briefly, PDOs were treated with different concentrations of either olaparib (0, 0.5, 1, 2, 5 and 10 μM) or cisplatin (0, 2, 5 10 and 20 μM) and CFA was used to quantify the survival fractions in 3D settings. To ensure the better visualization and counting of living cells, MTT was used to stain living cells within individual organoids pre‐harvesting (Fig. 7A). Compared with hormone‐sensitive ones, CRPC organoids were found to be clearly more sensitive to olaparib (Fig. 7B) and cisplatin (Fig. 7C), which indeed rationalizes the use of either olaparib or cisplatin as effective drugs in CRPC patients.

### 3.8 Enhanced pro‐metastatic signaling and migration in castration‐resistant cells

**Fig. 8:**
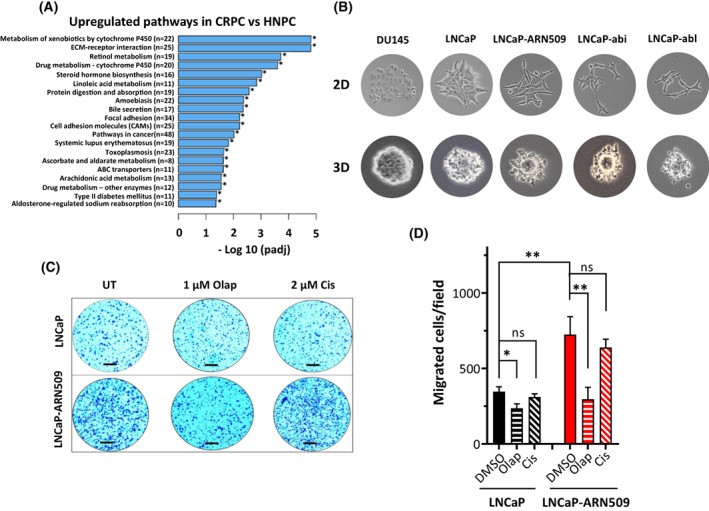
Pro‐metastatic pathways and migration are enhanced in castration‐resistant cells. (A) Gene ontology enrichment analysis of the significantly upregulated pathways [−log10 (adjusted *P*‐value)] in castration‐resistant cells showing upregulation in pro‐metastatic pathways. Significance: *P < 0.05. (B) Cell colony scattering assays were performed with the indicated cells by seeding cells at a low density and allowing them to form colonies in 2D or 3D cultures. Light microscopy images of the colonies were taken at random for each cell line. (C) Transwell migration assay: representative microscopic images of the indicated cells that migrated through the Transwell in the migration assay after treatment with 1 μM olaparib or 2 μM cisplatin for 36 h. UT, untreated control. Crystal violet was used to visualize cells. Scale bar: 200 μm. (D) Quantification of the experiments performed in (C). Shown are means ± SEM of four independent experiments (*n* = 3 for *cis*). Significance was calculated using the Mann–Whitney *U*‐test: *P < 0.05, **P < 0.01 vs. control. ns, not significant.

To date, metastatic CRPC remains incurable and the prognosis for these patients is poor. Therefore, it is important to have preclinical models to facilitate the identification of other treatment options for this disease setting. RNA‐SEQ analysis revealed more than 2000 upregulated genes in pooled castration‐resistant LNCaP sublines compared with the parental LNCaP cells (Fig. 2A). Interestingly, when gene ontology analysis was performed (Fig. 8A), we found that despite the fact that LNCaP cells were originally established from metastatic PCa, the most differentially upregulated molecular pathways in the resistant sublines were those related to metastasic progress, dissemination, including ECM receptor interaction, focal and cell adhesion molecules. This indicates that the metastatic potential might be further stimulated in the CR sublines. To investigate this issue, we monitored the ability of castration‐resistant clones to scatter outside the clones using a cell scattering assay. As illustrated in Fig. 8B, compared with the parental LNCaP cells, LNCaP‐ARN509, LNCaP‐abi and LNCaP‐abl cells displayed the typical scattering phenotype characterized by the loss of cell‐to‐cell contacts and drastic cellular elongation in both 2D and 3D culture settings. In contrast, DU145 cells, which are known to have a lower metastatic potential, showed no signs of cell scattering. Analysis of cell migration – an integral part of the metastatic cascade – using a chamber assay confirmed the enhanced invasive properties in the castration‐resistant LNCaP‐ARN509 cells compared with their hormone‐sensitive parental LNCaP cells as evidenced by a significantly higher number (twofold) of migrating cells in LNCaP‐ARN509 (346 ± 50.7 vs. 724.3 ± 188.4 migrated cells per field, *P* = 0.01). Interestingly, pretreatment with 1 μM olaparib significantly decreased the migrating cells both in LNCaP and LNCaP‐ARN509 castration‐resistant cells (*P* = 0.001) (Fig. 8C,D). In contrast, pretreatment with 2 μM cisplatin failed to reduce migration in LNCaP‐ARN509 cells. Notably, no change was seen in the proliferation or growth rate upon treatment with either olaparib or cisplatin for the entire 36 h of this experiment in either LNCaP or LNCaP‐ARN509 cells (Fig. S4). Together, these data reflect the ability of olaparib but not cisplatin to inhibit the metastatic behavior of CRPC cells.

## 4 Discussion

Compared with other tumor entities, translational research in PCa has lagged behind due to a lack of appropriate preclinical models. Few preclinical models accurately reflect the clinical and molecular variability seen in PCa patients, impeding the rational development of molecularly derived tailored treatment options. The techniques and models described in the current study are essential tools not only for bolstering the understanding of the drivers behind oncogenesis and how this affects the clinical course, but also to provide a rationale for alternative therapeutic targets for individual PCa patients.

Among the commonly used human PCa cancer models are cancer cell lines that are established from cancer patients. However, these do not represent PCa *in vivo*. Here we employed our previously reported CRPC‐induced sublines from the hormone‐sensitive PCa cell line LNCaP [31] and revealed compromised DSB repair efficiency and increased radiosensitivity in the hormone‐resistant clones than in the parental cells. WGS analysis did not show any evidence of genetic mutations in DSB genes. RNA‐SEQ demonstrated a different transcriptome in CRPC sublines with decreased expression of several HRR‐related genes such as *RAD51*. A lower RAD51 protein expression was also reported in the CRPC cells, resulting in HRR deficiency. In line with this finding, RAD51 foci recruitment at IR‐induced DSB sites was decreased twofold in CR sublines. These data pinpoint the HRR deregulation in CRPC and rationalize the use of PARPi for this subtype of the disease. In fact, the efficacy of different PARPi has been or is being tested for the treatment of mCRPC patients in several clinical trials. Most of these studies revealed that PARPi significantly improve tumor response in terms of disease control and overall survival for mCRPC patients with HRR mutations. Recently, nine clinical trials using PARPi in mCRPC have been analyzed in a meta‐analysis to test the benefit of PARPi in mCRPC patients [47]. The trials demonstrated that the magnitude of benefits from PARPi varies greatly between different HR‐defect subgroups, showing the most vigorous efficacy for PARPi in BRCA‐mutation carriers compared with patients who harbored no BRCA mutations. Furthermore, this analysis reported that BRCA2 mutations are likely the most effective mutations that predict the response to PARPi in PCa. Interestingly, a significant benefit in BRCA wild‐type tumors was observed, supporting the view that besides BRCA mutations, other non‐BRCA HRR‐related gene aberrations may also be used to predict the antitumor activity of PARPi. Hence, only using BRCA mutational status as a marker for PARPi sensitivity is inadequate, and it may miss a potentially larger proportion of responding patients. Following large‐scale cancer sequence analysis, mutations in other HR‐related genes such as CDK12, ATM and PALB2 were commonly found in mCRPC [11, 48, 49], and these non‐BRCA DNA repair genes could be used as alternative biomarkers to predict the sensitivity of PARPi. TOPARP‐A and B clinical studies by Mateo et al. [50, 51] provided evidence that mCRPC patients with other mutations in genes related to the HRR machinery also appear to benefit from PARP inhibitors.

A deep sequencing of all PCa patients would facilitate identifying the common genetic alterations with HRR to predict the benefit from PARPi. Despite the latest advances in the field of large‐scale sequencing analysis, it is still difficult to apply it in regular routine clinical work, as it is very costly and more importantly requires a previous knowledge about the role of each gene in HRR. In fact, there is only limited knowledge of the functional consequences of these mutations on HRR. Moreover, it is ultimately unclear why alterations in the same gene lead to a therapy response in one patient but not in another.

Here we present, in addition to the aforementioned *in vitro* cell lines, *ex vivo* preclinical models (*ex vivo* tumor slice and organoid cultures) that may help in detecting functional HRR defects to predict the response to PARPi. Furthermore, we present the rationale for the use of platinum‐based therapy such as cisplatin, which so far has not been routinely used in the treatment of CRPC but which has been reported to have some activity, especially in patients harboring HRR defects. This is in line with the previously published multicenter retrospective analysis showing antitumor activity for treatment with platinum‐based therapies in the cohort of CRPC patients with tumors harboring DNA repair gene aberrations [52]. Importantly, we believe that this is the first work that shows that olaparib but not cisplatin is able to impair the metastatic potential of PCa. Previously, we reported a functional *ex vivo* assay that enables the analysis of DSB formation and repair directly in tumor slice cultures from individual PCa patients [21]. Compared with tumor slices from HSPC patients, we clearly demonstrated here, in the CRPC slice cultures, an increase in the number of residual and thus unrepaired IR‐induced γH2AX and 53BP1 foci upon pretreatment with olaparib or cisplatin as evidenced by increased PiER and CisER indices, respectively. This was further confirmed using a modified approach to induce a CR phenotype through growing hormone‐sensitive tumor sample slices for several weeks in androgen‐depleted medium supplemented with CSS and abiraterone. Of note, there are some concerns about the applicability of the CR‐induction *ex vivo* approach in the clinical settings because of the relatively limited success rate in inducing CR (< 40% in our study) and the uncertainty as to whether the CR induction process represents the *in vivo* situation. Despite these concerns, this model confirmed the benefits of olaparib in patients pretreated with anti‐hormone therapy, especially abiraterone, which is in line with the results from the PROpel phase III study showing prolonged progression‐free survival for olaparib + abiraterone compared with abiraterone alone, irrespective of HRR status [53]. As a patient‐derived preclinical model, we further present here a very robust protocol for establishing organoids from PCa patients. Attempts to establish PCa organoids have been performed by other laboratories, but with lower overall success rates for longer/indefinite propagation and expansion. We could increase the success rate for the establishment of PCa organoids to 60–70% irrespective of hormone sensitivity state (HSPC and CRPC). This increased rate could be attributed to several factors such as ROCK inhibitor and epithelial growth factors, which enable the cells to adapt very quickly/more efficiently from tissue to culture conditions without inducing senescence as previously described in many studies [25, 26, 30]. Another explanation for our high success rate might be the efficient logistics, which enables the timely transport of freshly collected samples immediately from the operating theater to the lab, avoiding delays which may affect the efficiency of the lab‐based organoid formation. Tumor cell content has been confirmed in our established PCa PDO cultures by histopathological analysis. Also, PDOs have consistent IHC‐positive staining of the well‐established tumor marker AMACR and show histological similarities to their original primary tumors. In addition, evaluation of CpG‐rich global methylation revealed clustering of the organoids with other *in vivo* PCa datasets but not with other tumor entities or with normal prostate tissues. Given that PDOs enable the measurement of the direct effect on survival fractions, we demonstrated the increased sensitivity of CRPC organoids to olaparib or cisplatin compared with the organoids established from HSPC patients. Together, these data confirm a HRR‐deficient state of CRPC patients irrespective of distinct genomic alternations in known key players of HRR. It is important to note, however, that there is still space for future improvement for the PDO system presented here. For example, it is important to establish conditions that allow the establishment of microenvironmental elements such as blood vessels, immune cells and other stroma cells.

## 5 Conclusion

In conclusion, we present reliable preclinical models that allow for rapid functional testing and comparison of multiple individual drugs prior to *in vivo* analysis for example testing the presence of HRR deficiency in CRPC and response prediction to olaparib or cisplatin. This individual assessment of HRR functional capacity will enable us to improve future patient selection for personalized treatment approaches and thus increase the likelihood of response to PARP inhibitor therapy.

## Conflict of interest

The authors declare no conflict of interest.

## Author contributions

ME and WM designed the study concept and the experiments and wrote the paper. GvA, SJO‐H, CO, TM, AE, US, CP and KR collected and analyzed the clinical data. AE and US examined the methylome and performed the corresponding analysis; CM and MA performed the bioinformatics for WGS and RNA‐SEQ. SB‐R performed the pathological analyses of tumor and organoid samples SJO‐H provided drug‐resistant cell lines. ME and WM did artwork. All authors reviewed and edited the paper and have approved its final version.

### Peer review

The peer review history for this article is available at https://publons.com/publon/10.1002/1878‐0261.13382.

## Supporting information


**Fig. S1.** Whole genome profile differences between castration‐resistant cells and their parental sensitive counterpart LNCaP.
**Fig. S2.** Effect of olaparib or cisplatin on DSB‐repair in CR‐induced *ex vivo* PCa cultures.
**Fig. S3.** Effect of olaparib or cisplatin on DSB‐repair in freshly collected tumor tissues from hormone naïve or castration‐resistant PCa patients.
**Fig. S4.** Effect of 1 μM olaparib or 2 μM cisplatin on cell survival.

**Table S1.** Culture medium components for human prostate organoids.
**Table S2.** Clinical information and pathological parameters of donors for the established patient‐derived organoids.

## Data Availability

Sequence data have been deposited in the European Nucleotide Archive (ENA) at EMBL‐EBI under accession number PRJEB55017 (https://www.ebi.ac.uk/ena/browser/view/PRJEB55017). Generated raw data supporting the findings of this study are available from the corresponding author (WM) on request.
