# Supplementary material for: Preclinical patient‐derived modeling of castration‐resistant prostate cancer facilitates individualized assessment of homologous recombination repair deficient disease
Source: Mol Oncol. 2023 Mar 16;17(6):1129–47. doi: 10.1002/1878-0261.13382 (PMC10257417; doi:10.1002/1878-0261.13382)
Supplement: Supplementary file 1 — Fig. S1. Whole genome profile differences between castration‐resistant cells and their parental sensitive counterpart LNCaP. Fig. S2. Effect of olaparib or cisplatin on DSB‐repair in CR‐induced ex vivo PCa cultures. Fig. S3. Effect of olaparib or cisplatin on DSB‐repair in freshly collected tumor tissues from hormone naïve or castration‐resistant PCa patients. Fig. S4. Effect of 1 μM olaparib or 2 μM cisplatin on cell survival. [file MOL2-17-1129-s001.pdf]

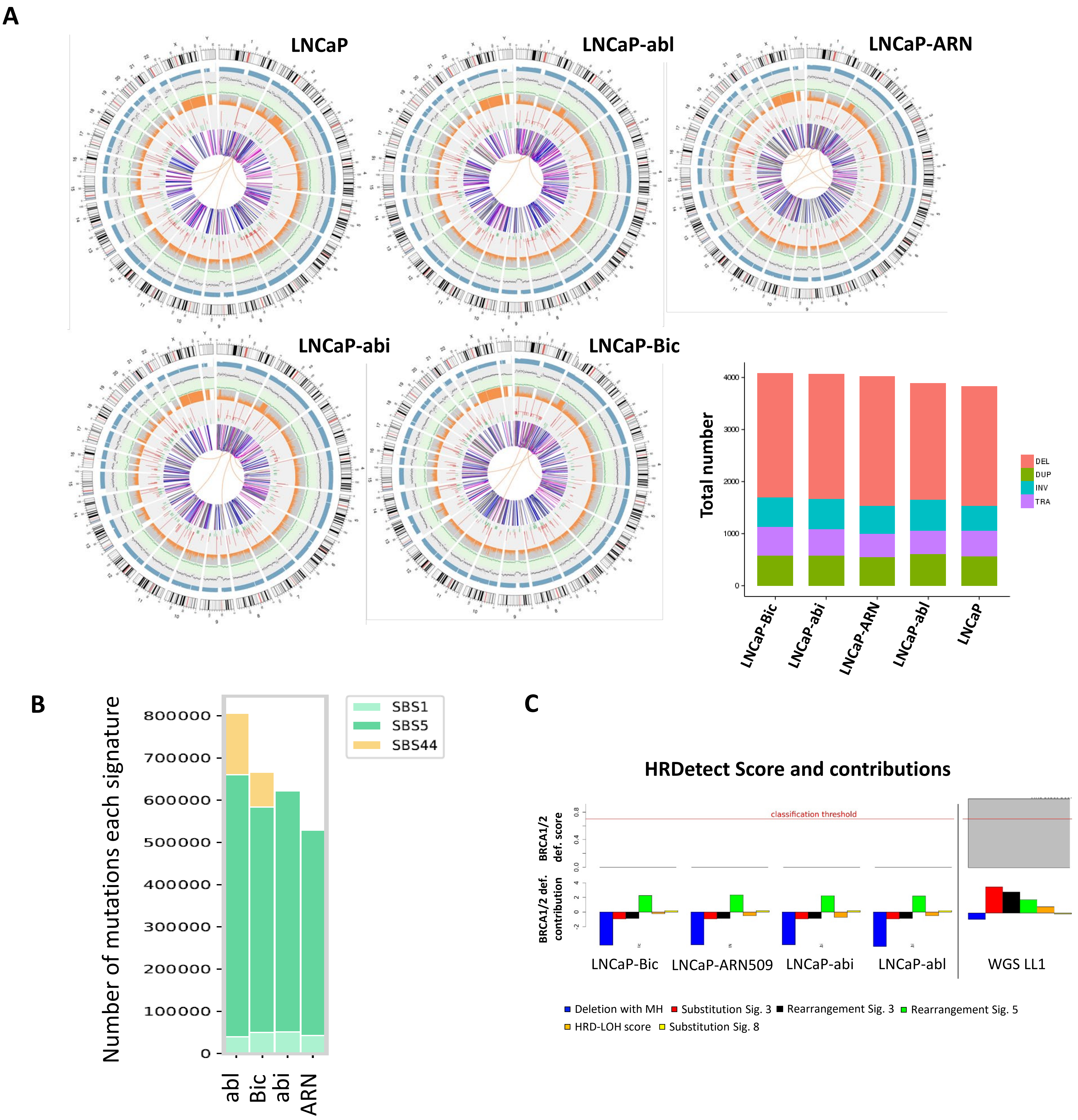

Supplementary Figure S1 Whole genome profile differences between Castration-resistant cells and their parental sensitive counterpart LNCaP. (A) Circos plots from outermost rings heading inwards: (i) The outer circle (the first circle) is chrome information. (ii) The second ring represents the read coverage in histogram style. A histogram is the average coverage of a 0.5Mbp region. (iii) The third ring represents indel density in scatter style. A black dot is calculated as indel number in a range of 1Mbp. (iv) The fourth ring represents snp density in scatter style. A green dot is calculated as snp number in a range of 1Mbp. (v) The fifth ring represents the proportion of homozygous SNP (orange) and heterozygous SNP (grey) in histogram style. A histogram is calculated from a 1Mbp region. (vi) The sixth ring represents the CNV inference. Red means gain, and green means loss. (vii) The most central ring represents the SV inference in exonic and splicing regions. TRA (orange), INS (green), DEL (grey), DUP (pink) and INV (blue). The size of genomic regions affected by CNVs in each sample. The x-axis represents samples, and the y-axis represents the total size of genomic regions affected by gains or losses (Mb). (B) Different single base substitutions (SBS) reported in the indicated CR sublines compared to their parental naïve cell line. (C) HRDetect scores measured by the BRCAness probability score on the y-axis and contributions by different mutational signatures for CR sublines compared to their parental naïve cell line. Publicly available data from a breast cancer cell line (HCC1395, HCC1395BL) and a matched control from the same patient were used as a positive control (WGS LL1). Signatures contributing to the HRDetect score were proportion of deletions with microhomology (blue), number of SBS3 (red) and SBS8 (yellow) mutations, number of mutations for rearrangement signatures 3 (black) and 5 (green) and loss of heterozygosity score (orange). The contributions are the normalized values of the features multiplied for the corresponding HRDetect logistic model coefficient. A BRCAness probability score > 0.7 was used to identify HRD.

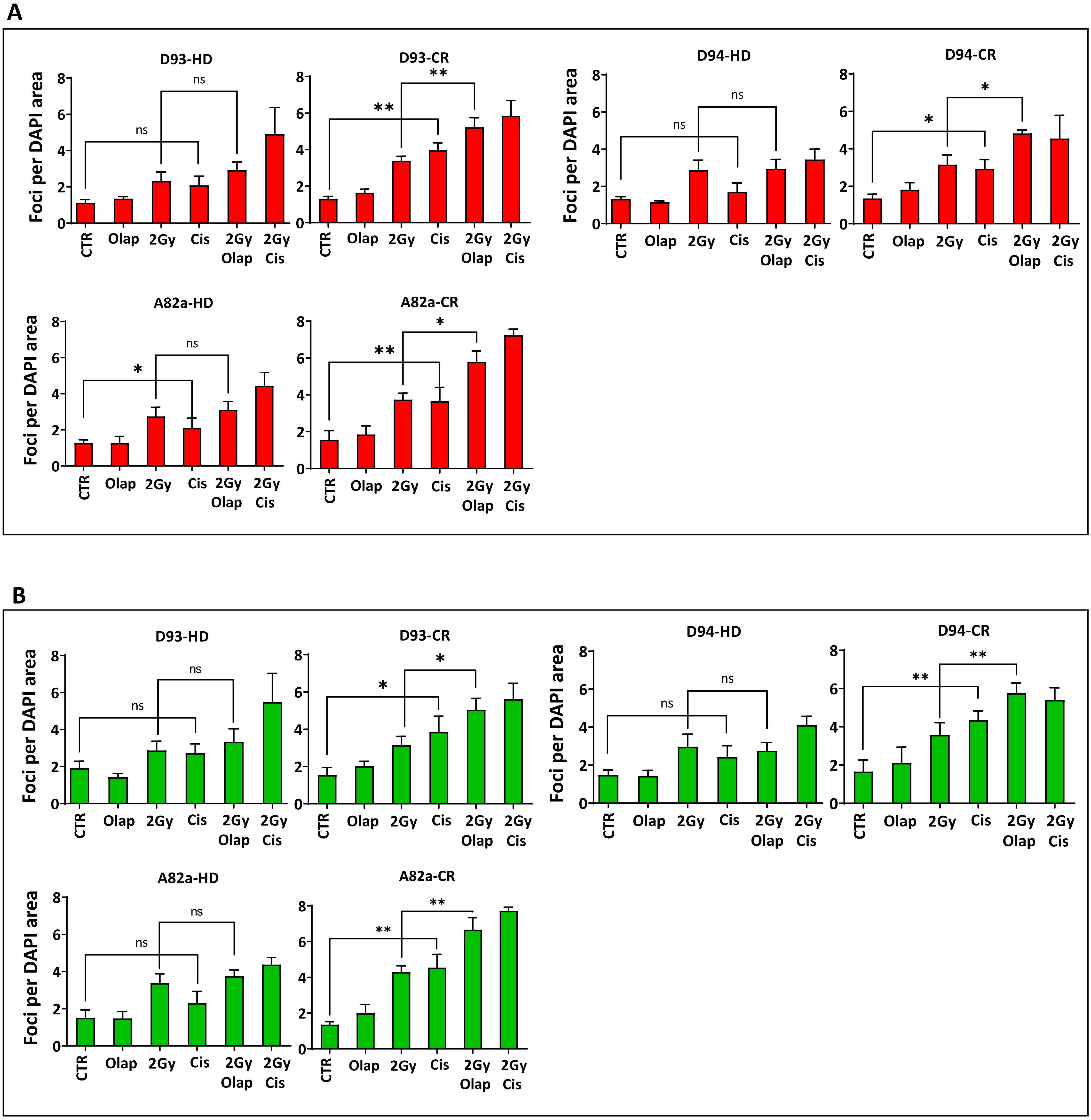

Supplementary Figure S2. Effect of Olaparib or Cisplatin on DSB-repair in CR-induced ex vivo PCa cultures. Number of (A) γH2AX or (B) 53BP1 foci were monitored in tumor slices cultured under hormone-dependent (HD) or castration resistance (CR) conditions 24h after the indicated treatments. Shown are the means ± SEM from at least three independent experiments. P-values were calculated using the Mann-Whitney U test. Significance is indicated as * for the $P < 0.05$, ** for P<0.001 and ns: not significant.

A

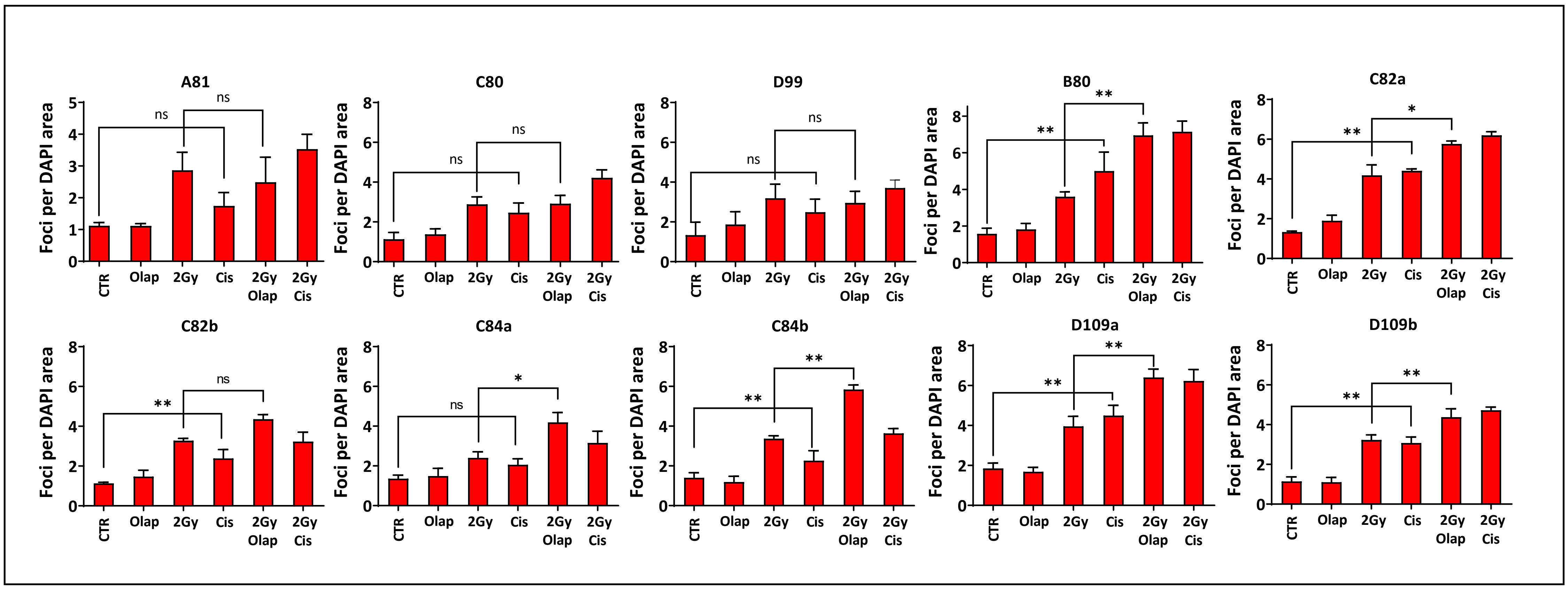

B

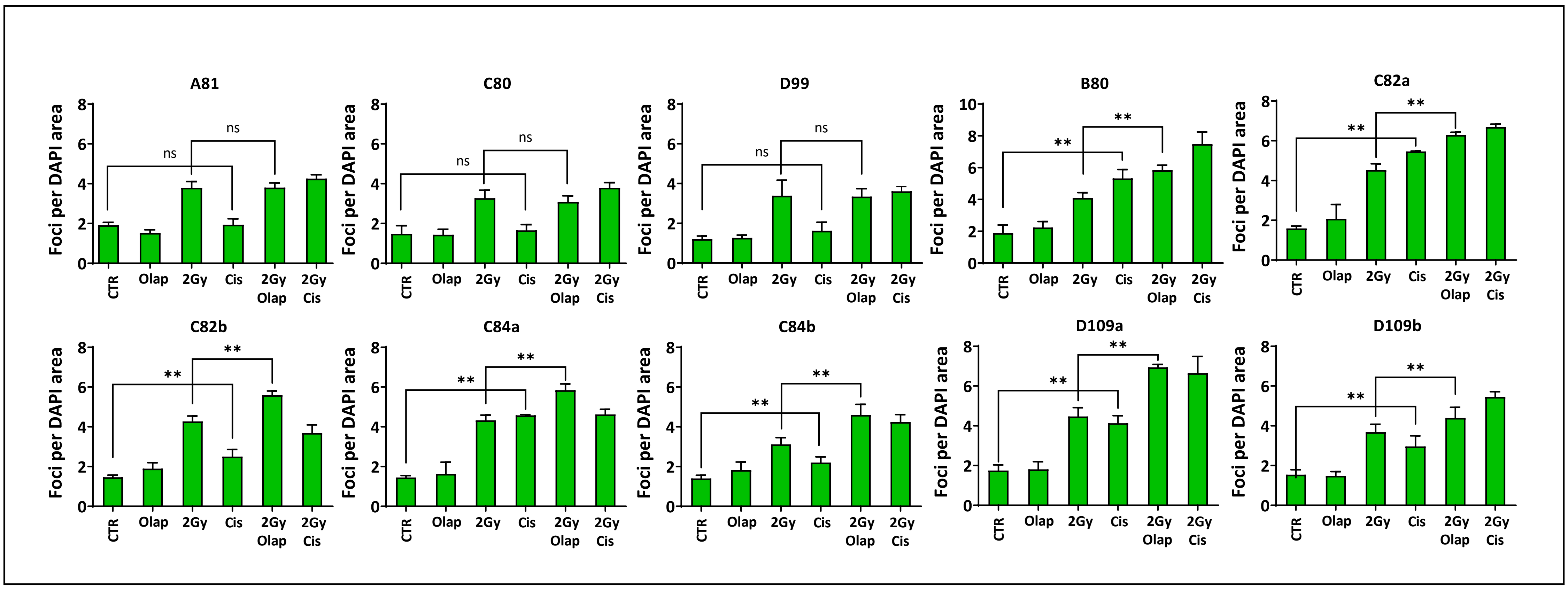

Supplementary Figure S3. Effect of Olaparib or Cisplatin on DSB-repair in freshly collected tumor tissues from hormone naïve or castration resistant PCa patients. Number of (A) γH2AX or (B) 53BP1 foci in tumor slices cultures 24h after the indicated treatments. Shown are the means ± SEM from at least three independent experiments. P-values were calculated using the Mann-Whitney U test. Significance is indicated as * for the $P < 0.05$, ** for P<0.001 and ns: not significant.

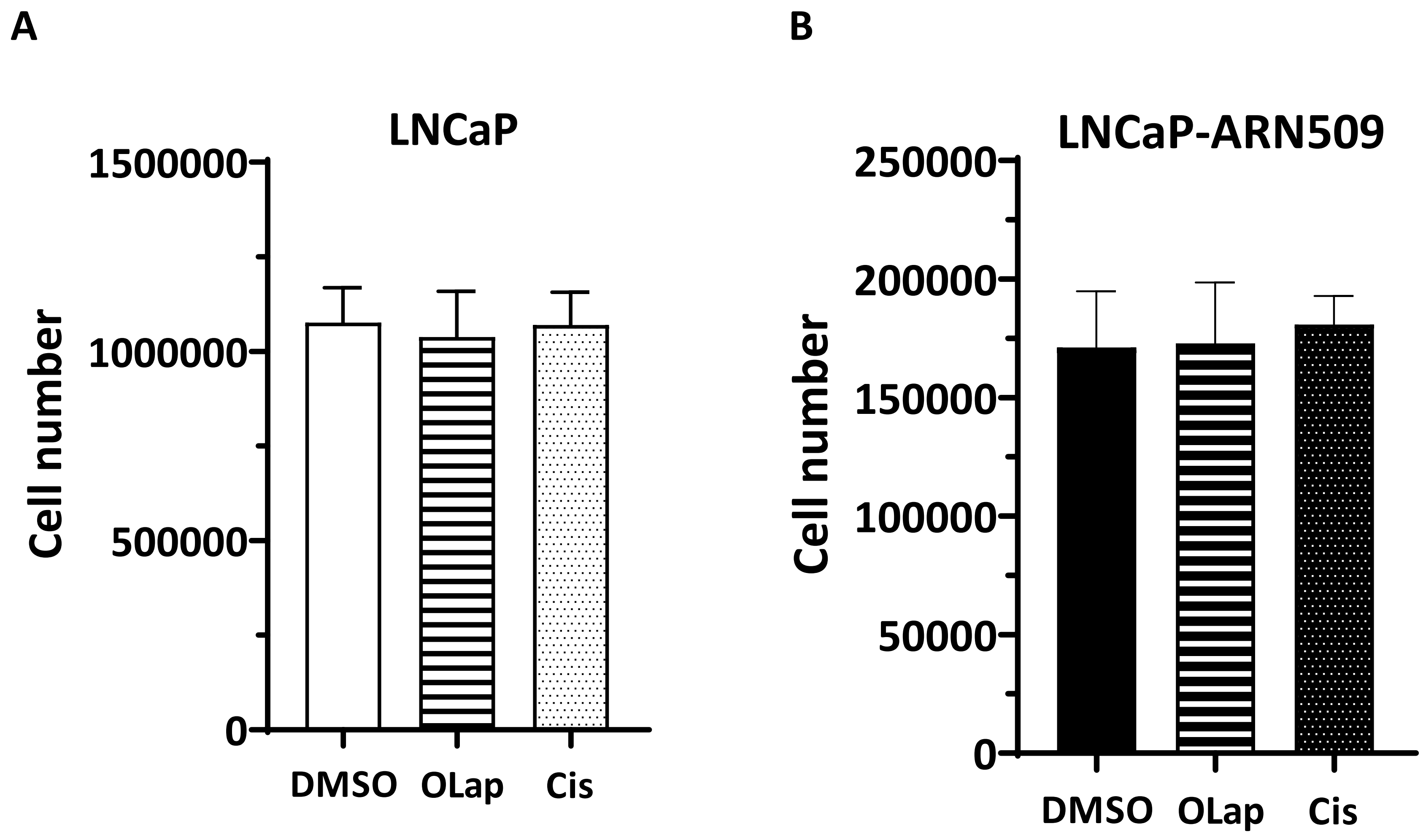

Supplementary Figure S4 Effect of 1μM Olaparib or 2μM cisplatin on cell survival. Cells were suspended in in FBS-free DMEM medium at concentrations of 2 $\times 10^5$ cells for (A) LNCaP and (B) LNCaP-ARN509. Seeded cells were then treated with DMSO, 1μM Olaparib (olap) or 2μM cisplatin (cis) and incubated for 36h before being counted.
