# Supplementary material for: Preclinical patient‐derived modeling of castration‐resistant prostate cancer facilitates individualized assessment of homologous recombination repair deficient disease
Source: Mol Oncol. 2023 Mar 16;17(6):1129–47. doi: 10.1002/1878-0261.13382 (PMC10257417; doi:10.1002/1878-0261.13382)
Supplement: Supplementary file 2 — Table S1. Culture medium components for human prostate organoids. Table S2. Clinical information and pathological parameters of donors for the established patient‐derived organoids. [file MOL2-17-1129-s002.pdf]

Table S1 Culture medium components for human prostate organoids

Compound	Supplier	Catalog #	Concentration
**Glutamax**	Thermo Sci	35050061	1x
**HEPES**	Thermo Sci	15630080	10 mM
**Pen/Strep**	Thermo Sci	15140122	1x
**B27 supplement**	Thermo Sci	17504044	1x
**N-acetylcysteine**	Sigma	A9165-5G	1.25 mM
**EGF**	PeproTech	AF-100-15	5 ng/mL
**Noggin**	PeproTech	120-10C	100 ng/mL
**R-spondin 1**	Conditioned medium		10%
**A83-01**	Sigma	SML0788	500 nM
**FGF10**	PeproTech	100-26	20 ng/mL
**FGF-7 (KGF)**	PeproTech	100-19	5 ng/mL
**Prostaglandin E2**	Sigma	P0409	1 µM
**Nicotinamide**	Sigma	N0636	5 mM
**SB202190**	Sigma	S7067	500 nM
**Y-27632 (ROCK inhibitor)**	Miltenyi	130-106-538	10 µM
**DHT**	Sigma	A8380	0.1nM for CRPC samples, 1nM for hormone-sensitive samples

Table S2. Clinical information and pathologic parameters of donors for the established patient-derived organoids

Organoid ID	Age* (years)	iPSA (ng/ml)	Neoadj. ADT	Tumor stage	Lymph node invasion	Surgical margin	Gleason score		CRPC feature**
							1ry Tumor	Organoid	
A79	59	7.9	No	pT3b	pN1 (1/6)	positive	4+5	4+5	No
A81	54	7.48	Yes	pT3a	pN0 (0/8)	positive	3+4	4+4	No
A82	63	8.4	No	pT3a	pN1 (3/19)	negative	4+3 with minor 5		No
A89	63	53,6	Yes	pT3b	pN1 (8/36)	positive	5+4	4+5	Yes
B80	60	41.5	No	pT3b	pN0 (0/12)	positive	4+3	4+3	Yes
C80	57	29	Yes	pT3b	pN0 (0/23)	negative	3+4 with minor 5		No
C82	68	11.9	Yes	pT3b	pN1(1/8)	positive	4+5		Yes
C84	58	106	No	pT3b	pN1 (12/26)	positive	5+4		Yes
D93	63	10.0	No	pT2c	pN0 (0/18)	negative	3+4	4+4	No
D94	71	92.12	Yes	pT3b	pN1 (2/12)	negative	4+5 with minor 3		Less sensitive to LHRH, sensitive to apalutamid
D99	66	49.7	Yes	pT3b	pN1 (2/27)	negative	4+5		No
D109	69	9.1	Yes	pT3b	pN0 (0/22)	negative	4+5 with minor 3	4+5	Yes
D110	68	142	Yes	pT3b	pN0 (0/29),	negative	4+3 with minor 5	4+4	Yes
D111	73	76,3	Yes	pT3a	pN1 (1/19),	positive	5+4	4+5	Sensitive to apalutamid

All tumor tissues are adenocarcinoma. Highlighted are patients from which organoids were established. iPSA: initial PSA at the time of tumor diagnosis by biopsy. ADT: Androgen deprivation therapy. * at the time of surgery ** testosterone > 20 ng/dL (0.7 nmol/L), inadequate PSA decrease or rapid clinical progress under (neoadjuvant) ADT
